# Parachute tricuspid valve: a systematic review

**DOI:** 10.1186/s13023-020-01561-y

**Published:** 2020-10-28

**Authors:** Shi-Min Yuan

**Affiliations:** Department of Cardiothoracic Surgery, The First Hospital of Putian, Teaching Hospital, Fujian Medical University, 389 Longdejing Street, Chengxiang District, Putian, 351100 Fujian Province China

**Keywords:** Cardiac surgical procedures, Congenital heart defect, Parachute tricuspid valve

## Abstract

**Background:**

A parachute tricuspid valve is a very rare congenital cardiac anomaly. Its morphological features and clinical implications have not been sufficiently described so far. The purpose of the present systematic review is to disclose the morphological and clinical characteristics of parachute tricuspid valve, and to discuss its diagnostic methods, treatments and patients’ outcomes.

**Main body:**

The Preferred Reporting Items for Systematic Reviews and Meta-analyses (PRISMA) statement guidelines were followed in this systematic review. Publications were systematically searched in the PubMed, Highwire Press, and the Cochrane Library databases. By comprehensive retrieval of the pertinent literature published between 1979 and 2019, 13 reports were collected with 14 patients recruited into this study. Their ages ranged from neonate to 52 years old with a median age of 23 years. Tricuspid valve regurgitation of a less-than-severe degree was seen in 6 (60%) patients, tricuspid valve stenosis was present in 3 (30%) patients and normally functioning tricuspid valve was noted in 1 (10%) patient. All patients had a single papillary muscle in the right ventricle. The chordae tendineae could be normal in length and thickness, or elongated, or shortened and thickened. Forty percent of the patients were asymptomatic or with only mild symptoms and did not need a surgical or interventional therapy, and 6 (60%) patients were indicated for a surgical/interventional treatment due to their severe presenting symptoms, associated congenital heart defects, and the resultant severe right ventricular inflow obstruction and (or) tricuspid stenosis. Patients’ outcomes varied depending on the substantial status of the patients with a survival rate of 70% and mortality rate of 30%.

**Conclusion:**

A few patients with a parachute tricuspid valve are asymptomatic or only with mild symptoms and a surgical or interventional treatment is not required. The surgical/interventional indications for parachute tricuspid valve patients are their severe presenting symptoms, associated congenital heart defects, and the resultant severe right ventricular inflow obstruction and (or) tricuspid stenosis. The survival rate of this patient setting is satisfactory.

## Introduction

An atrioventricular valve anomaly caused by a single papillary muscle with unifocal attachment of the chordae tendineae was firstly described by Swan et al. [1] in 1949. This anomaly was termed as "parachute" by Schiebler et al. [2] in 1961. Schiebler et al. [2] and El Sayed et al. [3] observed a parachute anomaly affecting the right-sided atrioventricular valve in patients with corrected transposition of the great arteries. The right atrioventricular valve in such patients is actually the morphological mitral valve, whereas the left-sided atrioventricular valve is the tricuspid valve. Thus it should be categorized as parachute mitral valve. Parachute tricuspid valve (PTV) is a very rare congenital lesion. Due to its rarity, the anatomical features and clinical implications of PTVs remain uncertain. The purpose of this article is to describe the anatomical and clinical features of PTV, and discuss the management, surgical indications and outcomes of the patients.

## Materials and methods

**Fig. 1:**
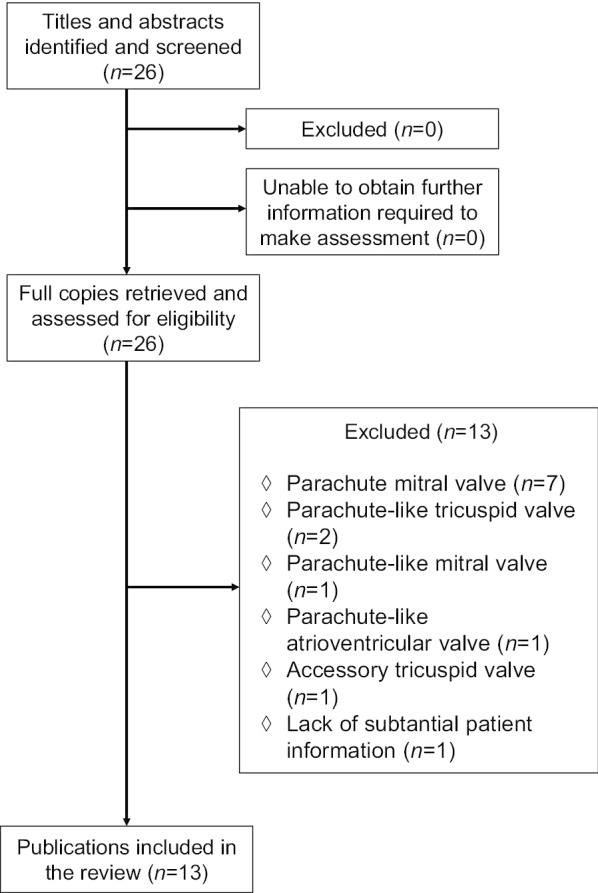
A flow chart of literature retrieval

The Preferred Reporting Items for Systematic Reviews and Meta-analyses (PRISMA) statement guidelines were followed in this systematic review. Publications were systematically searched in the PubMed, Highwire Press and the Cochrane Library databases. The period of study was from 1979 to 2019. The MeSH terms and keywords used to identify articles included “parachute tricuspid valve”, “supravalvular tricuspid ring”, “single papillary muscle”, “congenital tricuspid stenosis”, “tricuspid valve repair”, and “tricuspid valve replacement”. The screening of the bibliographic references helped in completing the literature retrieval. Twenty-six articles were found related to the topic and keywords in the literature search; and 13 articles, which met the inclusion criteria during preliminary assessment, were included in this review. The inclusion criteria were publications of any type (clinical research, case series, or case report) on PTV patients at all ages including children (age < 18 years old) and adults (age ≥ 18 years old) with substantial patient information for statistical analysis. The exclusion criteria were: parachute mitral valve (*n* = 7), parachute-like tricuspid valve (*n* = 2), parachute-like mitral valve (*n* = 1), parachute-like atrioventricular valve (*n* = 1), accessory tricuspid valve (*n* = 1), and lack of substantial patient information (*n* = 1). A flow chart of literature retrieval was shown in Fig. 1.

The data independently extracted from each study were the study population, demographics, morphology and function of the tricuspid valve, associated congenital heart defects, surgical indications, management and patients’ outcomes.

The measurement data were expressed in mean ± standard deviation. The categorical variables were expressed by frequency and percentage, and were compared by Fisher's exact test. *P* < 0.05 was considered statistically significant. An IBM SPSS Statistics version 22.0 was used to perform the statistical analyses.

## Results

In total 13 articles were collected with 14 patients involved [4–16]. Patient information in detail was listed in Table 1. As a result, 12 articles were single case reports and one article was a report inclusive of 2 cases. No clinical research or large case series were included. There were 9 (64.3%) adult and 5 (35.7%) pediatric patients (*χ*^2^ = 2.3, *P* = 0.257). Patient age was not described for one case [12]. For the remaining 13 cases, ages ranged from neonate to 52 years old with a median age of 23 years. Their mean age was 21.3 ± 15.7 (range, 0–52; median, 23) years (*n* = 13). Gender was known for 11 patients: 6 (54.5%) were male and 5 (45.5%) were female patients (*χ*^2^ = 0.2, *P* = 1.000).

**Table 1:** Detailed patient information

Year	Author	Age (year)	Sex	Papillary muscle	Diagnotic method	Associated anomaly	Enlarged heart chamber	TV	Tricuspid chord	PG (systolic/diastolic/mean) (mmHg)	Treatment	Follow-up (month)	Outcome
2017	Alimi and Fazlinezhad [5]	52	F	Single, calcified	TTE, 3D-TEE	ASD	RA, RV	TR (moderate to severe)			Device closure of ASD	2	Improved (mild RV enlargement with mild to moderate TR)
2017	Alimi and Fazlinezhad [5]	30	F	Single, calcified	TTE, 3D-TTE	No		TR (mild to moderate)			Follow-up		No change
1979	Ariza et al. [6]	Neonate				Tetralogy of Fallot with pulmonary atresia	RA	TS					
2011	Demirkol et al. [7]	21	M	Solitary	TTE, 3D-TTE, 3D-TEE, computed tomographic angiography		Pear-like RA	Funnel-shaped, TR (trivial)		7/3/?			
2012	Demirkol et al. [8]	24	M	Bifid single	TTE, 3D-TTE, TEE, 3D-TEE, CTA	ASD	RV	Doming shaped, TR (trivial)	Elongated	4/3/?	Surgical closure of ASD		Recovered
1997	Godart et al. [9]	Neonate (she grew up to 2 years)	F	Single anterior papillary	Echocardiography	C-TGA, VSDs, small RV (TV annulus 9 mm), hypoplastic aortic arch, coarctation, PDA, s/p coarctation repair + arch plasty + banding, s/p debanding, PAH		TV obstruction (annular ring), TS		?/?/18	Annular ring resection + TVR		Died of postoperative septic shock
2016	Gupta et al. [10]	0.625	M		TTE	ASD, VSD (perimembrane)		TV stenosis		14/7/?	VSD closure + chord transition (to support the free edge of the neoseptal leaflet) + ASD closure	4	Complicated: *Escherichia* coli pneumonia, recovered
2013	Kurtul et al. [11]	33	M		TTE	Parachute MV, VSD (perimembrane), PAH (44 mmHg), anomalous origin of the circumflex artery from the right sinus of Valsalva	RV, LA	Normal function			Transcatheter closure of VSD		Recovered
1980	Maitre Azcarate [12]			Single from apex	Autopsy	Cor triatriatum		Anterior and posterior leaflets were fused					Died
2006	Marwah et al. [13]	21	F	Single		ASD, VSD (perimembrane)	RV	Annulus 22 mm, mild prolapse of septal leaflet, TS, mild prolapse, normally functioning			No: planned but not yet		Unchanged
1979	Milo et al. [4]	0.1 (he grew up to 6 years 8 months old)	M	Single	Autopsy	Double outlet right ventricle, straddling mitral valve, severe pulmonary outflow tract obstruction, and posterior inlet VSD	RV		Shortened and thickened chordae tendineae supporting the valve		Stadding mitral valve repair + VSD closure		Died of cardiac deterioration at 24 h
2015	Mohan et al. [14]	23	F	Single	TTE	Parachute mitral valve, corrected transposition, right-sided aortic arch, valvular pulmonary stenosis, bicuspid aortic valve		TR (mild)	Normal length and thickness of the tendinous chords		No (mild symptom)		Unchanged
2012	Mohan et al. [15]	30		Single short	TTE, multislice cardiac CT	ASD, PAH		TR	Shorter	8/4/?			
2010	Uçar et al. [16]	25	M	Single	TTE	VSD							

*3D* three-dimensional, *ASD* atrial septal defect, *CT* computed tomography, *CTA* computed tomographic angiography, *C-TGA* corrected transposition of the great arteries, *F* female, *LA* left atrium, *M* male, *MV* mitral valve, *PAH* pulmonary artery hypertension, *PDA* patent ductus arteriosus, *PG* pressure gradient, *RA* right atrium, *RV* right ventricular, *s/p* status post, *TEE* transeaophageal echocardiography, *TR* tricuspid regurgitation, *TS* tricuspid stenosis, *TTE* transesophageal echocardiography, *TV* tricuspid valve, *TVR* tricuspid valve replacement, *VSD* ventricular septal defect

In one report, patient’s symptom was not mentioned [12]. In the remaining 13 patients, 3 (23.1%) were asymptomatic and 10 (76.9%) were symptomatic (*χ*^2^ = 7.5, *P* = 0.017). The major symptoms of the 10 symptomatic patients were palpitations [5, 7], heart failure [9, 10], dyspnea [4] and peripheral edema [6].

A cardiac murmur was audible in 11 patients. Actually a systolic murmur was audible in all 11 patients. In 10 patients, the murmur was audible along the sternal border, and in 1 patient, it was heard in the left intercostal space [8]. In one of the patients, an additional diastolic murmur was heard at the apex [4]. A fixed split of the second heart sound was heard in 3 (27.3%) patients [5, 13, 14].

The electrocardiographical results were described for 7 patients: 2 (28.6%) patients had a normal sinus rhythm [5, 7], and 5 (71.4%) patients had an abnormal electrocardiogram including right bundle branch block (*n* = 1) [8], conduction defects (*n* = 1) [6], left ventricular hypertrophy (*n* = 1) [9], ST-T changes (*n* = 1) [5], and north-west axis (+ 150°), right atrial enlargement and prominent R wave in the V_1_ lead (*n* = 1) [14].

The diagnostic methods of PTV were described in 12 patients: it was diagnosed by transthoracic echocardiography (*n* = 5, 41.7%) [9–11, 14, 16], by both transthoracic and three-dimensional transesophageal echocardiography (*n* = 2, 16.7%) [5], by transthoracic and transesophageal echocardiography, three-dimensional transthoracic and transesophageal echocardiography and computed tomographic angiography (*n* = 2, 16.7%) [7, 8], by transthoracic echocardiography and multislice cardiac computed tomography (*n* = 1, 8.3%) [7], and by autopsy (*n* = 2, 16.7%) [4, 12].

In 6 (42.9%, 6/14) patients, cardiac chamber enlargement was noted: 4 (66.6%) patients had one cardiac chamber enlargement (right ventricular enlargement (*n* = 3) [4, 8, 13] and right atrial enlargement (*n* = 1) [6]), and 2 (33.3%) patients had two cardiac chamber enlargement (right atrial and right ventricular enlargement (*n* = 1) [5] and left atrial and right ventricular enlargement (*n* = 1) [11]).

All patients had a single papillary muscle in the right ventricle. The papillary muscle was described as: calcified (*n* = 2) [5], short (*n* = 1) [15], from the apex (*n* = 1) [12] and a single anterior one (*n* = 1) [9].

The chordae tendineae were narrated for 4 patients: they were with normal length and thickness (*n* = 1) [14], elongated (*n* = 1) [8], shorter (*n* = 1) [15] and shortened and thickened (*n* = 1) [4].

The morphology and (or) function of the tricuspid valve were described for 12 patients. In one patient, the anterior and posterior leaflets were fused, and the functional status of the tricuspid valve was not given [12]. In another patient, the conditions of one tricuspid valve were confusely narrated as “annulus 22 mm, mild prolapse of the septal leaflet, tricuspid stenosis and functionally normal” [13]. This case was excluded from the statistical analysis of tricuspid valve function. In the remaining 10 patients, 6 (60%) patients had tricuspid regurgitation (it was trivial (*n* = 2, 33.3%) [7, 8], mild (*n* = 1, 16.7%) [14], mild-to-moderate (*n* = 1, 16.7%) [5] and moderate-to-severe [5] (*n* = 1, 16.7%)), 3 (30%) had tricuspid stenosis [6, 9, 10] and 1 (10%) had a normally functioning tricuspid valve [11]. The systolic tricuspid pressure gradient was 8.3 ± 4.2 mmHg and the mean tricuspid pressure gradient was 4.3 ± 1.9 mmHg [7, 8, 10, 15].

Of the 14 patients, 12 (85.7%) patients had one or more associated congenital heart defects, including atrial septal defect [5, 8, 10, 13, 15], ventricular septal defect [10, 13, 16], cor triatriatum [12], corrected transposition of the great arteries [9, 14], tetralogy of Fallot and pulmonary atresia [6] and parachute mitral valve and anomalous origin of the circumflex branch from the right sinus of Valsalva [11]; and 2 patients had an isolated PTV (14.3%). PTVs associated with other cardiac malformations were significantly more than isolated PTVs (*χ*^2^ = 14.3, *P* < 0.001). Moreover, 3 (21.4%) patients had pulmonary artery hypertension [9, 11, 15].

The treatments were not described for 4 patients. Of the remaining 10 patients, a surgical/interventional treatment was required in 6 (60%) patients: tricuspid valve operation in 2 (33.3%) patients (annular ring resection plus tricuspid valve replacement [9], and chord transition [10]), and surgical/interventional repair of other associated intracardiac anomalies in 5 (83.3%) patients (the patient receiving chord transition had simultaneous atrial and ventricular septal defect closures [10]) including device closure of atrial septal defect [5, 11], surgical closure of atrial septal defect [8], chord transition (to support the free edge of the neoseptal leaflet) plus atrial and ventricular septal defect closures [10] and straddling mitral valve repair plus ventricular septal defect closure [4]. In addition, 4 (40%) patients were on a follow-up only due to mild clinical symptoms. There was no significant difference in the prevalence between surgical/interventional and non-surgical/interventional patients (*χ*^2^ = 0.8, *P* = 0.656).

The surgical/interventional indications for the 6 patients were an associated congenital heart defect (*n* = 4, 66.7%) [5, 8, 11, 14], congestive heart failure and associated congenital heart defect (*n* = 1, 16.7%) [10] and tricuspid stenosis and tricuspid annular ring (*n* = 1, 16.7%) [9].

Patients’ outcomes were reported for 10 patients: 2 (20%) patients recovered fully [8, 11], 1 (10%) patient improved with mild-to-moderate tricuspid regurgitation [5], 1 (10%) patient was complicated with *Escherichia coli* pneumonia and finally recovered after treatment [10], 3 (30%) patients remained unchanged [5, 13, 14] and 3 (30%) patients died [4, 9, 12]. The death causes of the 3 deceased patients were postoperative circulatory deterioration [4], postoperative septic shock [9], and an unspecified death cause [12], respectively. The two postoperative deaths occurred in two children at the age of 2 and 6 years 8 months, respectively (Table 2).

**Table 2:** Surgical/interventional treatments and prognosis of patients with parachute tricuspid valve

Treatment	Treatment technique	Case number	Patient age at operation (year)	Prognosis
Intervention	Transcatheter closure of ASD [5]	1	52	Improved
	Transcatheter closure of VSD [11]	1	33	Recovered
Surgery	Surgical closure of ASD [8]	1	24	Recovered
	VSD closure + chord transition (to support the free edge of the neoseptal leaflet) + ASD closure [10]	1	0.625	Complicated: *Escherichia* coli pneumonia, recovered
	Annular ring resection + TVR [9]	1	2	Died of postoperative septic shock
	Stadding mitral valve repair + VSD closure [4]	1	6.67	Died of cardiac deterioration at 24 h

*ASD* atrial septal defect, *TVR* tricuspid valve replacement, *VSD* ventricular septal defect

## Discussion

In 1979, Milo et al. [4] firstly reported a case of PTV associated with a straddling mitral valve and doubled outlet right ventricle in a baby. He was performed an operation at the age of 6 years 8 months but died soon after the operation. PTV is a very rare congenital cardiac anomaly. It is considered to have been underestimated, as the anatomical features are often sheltered by the associated congenital anomalies [12]. Its prevalence appears to be significantly lower than that of parachute mitral valve [4].

The anatomical features of the atrioventricular valve caused by two asymmetric papillary muscles are termed as a parachute-like valve [17]. The true type of PTVs should be distinguished from the parachute-like valve. In one report, a case of true PTV was mistaken as a parachute-like one [5]. This case was enrolled into this study as a case of true PTV.

Patients with a PTV can be asymptomatic if the valve function is normal or only with mild valve dysfunction [16]. However, in some patients, PTVs may cause tricuspid stenosis and (or) regurgitation. Patients with severe tricuspid stenosis may present with congestive heart failure [10].

A PTV can be visualized in two- and three-dimensional transthoracic and transesophageal echocardiography. A transgastric view is preferred to reveal the features of a PTV, including a solitary papillary muscle, pear-like right atrium and chordal redundancy. Multidetector computed tomography can also reveal the feature of a PTV with a single papillary muscle as well as its attachment site [8].

It has been reported in the literature that PTV was associated with right heart obstructions, such as tetralogy of Fallot [6] and double outlet right ventricle [4]. Atrial and (or) ventricular septal defects were often present in PTV patients. Parachute deformity of both mitral and tricuspid valves were reported in 2 patients [11, 14]. Mohan et al. [14] summarized 7 cases of PTV patients from the literature, and found the ratio of complex and solitory PVT was 4:3 (1.3:1). Tricuspid stenosis was less significant in PTV patients [5]. Transthoracic/transesophageal echocardiography was a usual diagnostic technique confirming the morphological features of PTV including single papillary muscle, doming shaped chords and pear-shaped right atrium [7]. Three-dimensional transesophageal echocardiography and computed tomography angiography also confirmed the features of PTVs [7]. Patients with a PTV often underwent a surgical treatment of the associated cardiac defects, and an operation for the tricuspid valve per se was usually not demanding unless there was severe tricuspid stenosis. Gupta et al. [10] reported their complex tricuspid valvuloplasty including single papillary muscle mobilization, chord transfer, anteroseptal commissure creation and regional annuloplasty for their PTV patient with a unicuspid tricuspid valve. Moreover, in PTV patients, transcatheter atrial septal defect closure could reduce the severity of tricuspid regurgitation as a result of reduction of tricuspid annular dimension and right ventricular volume overload.

The present study revealed that the morphological changes of the tricuspid valve of PTV patients could be functionally normal (10%), stenosed (30%), or regurgitant (60%). In line with what was reported in the literature, due to the small number of tricuspid stenosis cases and the mildly regurgitant tricuspid valve, the demanding of tricuspid valve operation was less likely in this patient setting. The tricuspid chords in patients with a PTV varied morphologically significantly from normal to shortened or elongated. In the present study, PTV associated with other cardiac anomalies was seen in 85.7% of the patients, while isolated PTV accounted for 14.3% with a ratio of 6:1, significantly exceeding the value reported in the literature. Surgical treatment was performed in 60% patients with a tricuspid valve operation in one-third and repair of associated cardiac defects in two-thirds of the patients. In 40% patients, only follow-up is advised due to no or mild symptoms. The prognoses of the patients were good with a survival rate of 70%.

As it was evidenced by the present study, when PTV patients became symptomatic, associated with additional congenital heart defects, or right ventricular inflow obstruction and (or) severe tricuspid stenosis, they were indicated for a surgical/interventional treatment [16]. Patients’ outcomes vary from case to case depending on the substantial status of the patients [8]. The present article proved that 60% of patients warranted a surgical/interventional treatment, and the mortality rate was 30%.

The study materials were based on some case reports, and this situation might bring about possible publication biases at an outcome level, and which was considered a major drawback of the study. In spite of the limitations, the value of the work still exists.

## Conclusion

PTV is a very rare congenital cardiac anomaly. Minority of patients are asymptomatic or only with mild symptoms and do not need a surgical/interventional therapy. The surgical/interventional indications for PTV patients are their severe presenting symptoms, associated congenital heart defects, and the resultant severe right ventricular inflow obstruction and (or) tricuspid stenosis. Patients’ outcomes vary depending on the substantial status of the patients.

## Data Availability

Not applicable.
